# Imaging acoustic sources through scattering media by using a correlation full-matrix filter

**DOI:** 10.1038/s41598-018-34039-w

**Published:** 2018-10-23

**Authors:** Wei Rui, Chao Tao, Xiaojun Liu

**Affiliations:** 1 Laboratory of Modern Acoustics, Department of Physics, Collaborative Innovation Center of Advanced Microstructures, Nanjing University, Nanjing, 210093 China; 2 Shenzhen Research Institute of Nanjing University, Shenzhen, 51800 China

## Abstract

In the inhomogeneous medium, acoustic scattering is always a fundamental challenge for photoacoustic imaging. We implement a correlation full-matrix filter (CFMF) combing with a time reversal operator to improve the imaging quality of acoustic sources in complex media. The correlation full-matrix filtering process extracts the direct wave component from the detected signal and preserve all the useful information at the same time. A location factor is considered in the time reversal operator to compensate for the image distortion and false contrast caused by the limited-view detection. The numerical simulations demonstrate that the proposed approach can perform good imaging quality with the higher image signal-noise ratio and better resolution in an acoustic scattering environment. This scheme might be applied to improve the photoacoustic imaging for inhomogeneous biological tissues.

## Introduction

Probing or imaging an acoustic source in an acoustic scattering environment is a fundamental challenge in disordered systems theory^1–4^ but highly desirable. It has many significant applications, such as geological prospecting^5^, underwater acoustic detection^6^, biomedical imaging^7–12^, and so on^13^. For example, photoacoustic imaging is essentially a process of probing sound sources based on passively received ultrasound signals. It combines the advantages of the rich contrast from optical imaging with the high spatial resolution in deep tissue from ultrasonography. Optical absorbers in the region of interest (ROI) are illuminated by a pulse laser and emit ultrasound waves as a result of the photoacoustic effect. Then the acoustic waves propagate through the scattering layer and are received by the ultrasonic transducer array to reconstruct the image. Photoacoustic imaging has great application prospects in biomedical imaging^7–11^.

Classical methods usually locate or image the acoustic sources by achieving coherent beamforming, which utilizes the deterministic signal phases in the direct wave component. However, a scattering contribution always exists when an acoustic wave is propagating through a random inhomogeneous medium. The coherence of the signals will be broken by the randomness of the scattering, which results in the appearance of speckles and even makes imaging failure. Acoustic scattering caused by an inhomogeneous medium is a nightmare for classical imaging techniques. It is generally sufficient to image the medium correctly by choosing an appropriate frequency doming where scattering can be neglect for its low strength. But in other situations, scattering can be so strong that there is no longer a direct relation between travel time and depth. As a consequence, the wave loses its coherent and coherent beamforming fails. Therefore, it is necessary to reduce the influence of scattering wave on probing acoustic target.

Over the years, there has been considerable effort to overcome the limitation of probing acoustic sources in the medium with inhomogeneous acoustical properties, including the statistical reconstruction method^14^, time reversal method^15,16^, coherence factor optimization^17^, interferometry method^18^, and so on^19–22^. Nevertheless, these methods are limited by specific conditions (need to know some prior properties of tissue inhomogeneity). To provide a universal scheme for more general situations, a random matrix theory has been put forward as a new way for sound wave analysis and the scattering medium is regarded as one realization of a random process^23^. The random matrix theory has shown great potential in acoustic backscattering imaging^23–25^, optical imaging^26,27^, and telecommunication^28–30^ in complex media. In wave physics, especially for the active detection, the matrix form is particularly suitable for describing the wave transmission and reveal the inherent deterministic coherence of the acoustic signal in the inhomogeneous medium effectually^23^.

Recently, we have generalized the random matrix theory to passive probing of acoustic sources in complex media^31^. The intrinsic coherence of the direct wave can be revealed in a matrix form by investigating the passively received wide-band ultrasound waves which propagate through the scattering media. On account of the correlation of direct wave, we proposed a matrix filter to separate the direct waves from the scattering waves. Benefitting from this correlation filter, the imaging quality of acoustic sources is significantly improved. However, this correlation filter still has some defects that need further improvement. The operations of matrix rotation and transformation make the partial acoustic information not fully utilized during matrix filtering process. Inadequate utilization of the matrix information results in the reduction of field-of-view and the image distortion of the acoustic target.

In this study, we propose a correlation full-matrix filter to restore the imaging area and improve the imaging quality. On the one hand, we improve the rotation and filtering operations during the matrix filtering to restore the imaging area and make full use of all the useful information in the detected signals. On the other hand, a location factor is proposed to compensate for the intensity unbalance in multi-targets imaging. Both simulations and experimental results verify the superior performance of the proposed matrix filter.

## Results

### Schematic of the scenario considered in this study

**Figure 1:**
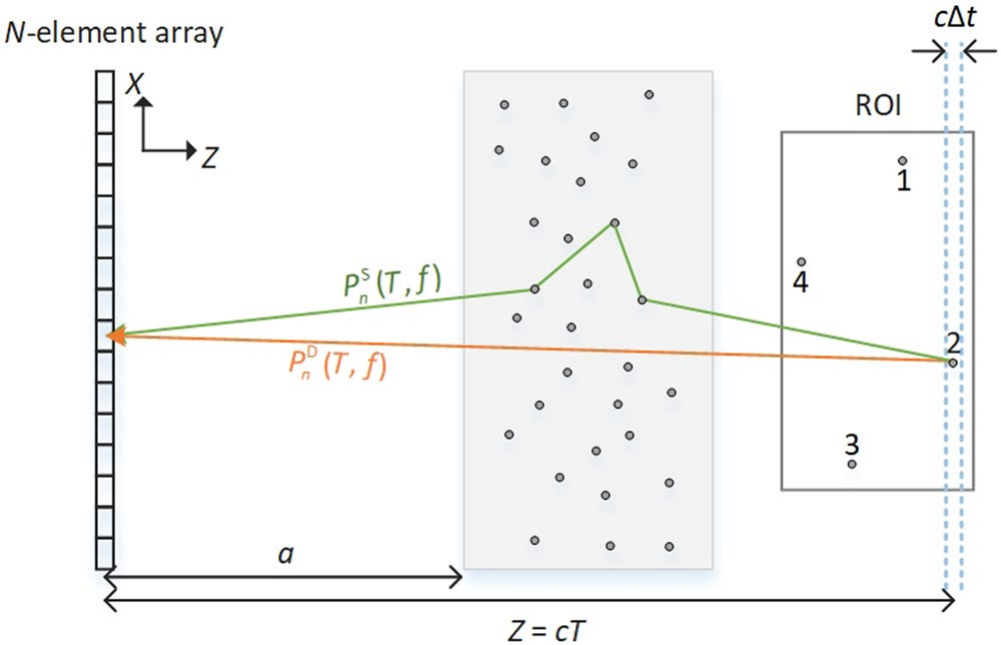
The schematic of the scenario to imaging through acoustic scattering layer. The signals are generated from the region of interest (ROI), propagate through the scattering layer (gray region), and are finally detected by the transducer array. The detected signals in the time window [*T *− ∆*t*/2, *T *− ∆*t*/2] contain two components: **P**^D^(*T*, *f* ) and **P**^S^(*T*, *f* ), around the time of flight *T* and at the frequency *f*. ${P}_{n}^{{\rm{D}}}(T,f)$ is one of the direct waves emitted directly from the object located at a depth of *Z* = *cT*, while ${P}_{n}^{{\rm{S}}}(T,f)$ is one of the scattering waves.

As shown in Fig. 1, four acoustic sources (marked as sources 1~4) with a diameter of *d* = 0.8 mm are placed in the ROI behind a scattering layer. Irradiated by the pulse laser, these four targets in the ROI emit ultrasonic waves (central frequency of 2.0 MHz, bandwidth of 1.26~2.68 MHz). The locations of the four acoustic sources 1~4 are (15 mm, 82 mm), (−5 mm, 87 mm), (−15 mm, 77 mm) and (5 mm, 72 mm), respectively. For the acoustic sources, the speed of sound is *c*_s_ = 5200 m/s and the density is *ρ*_s_ = 7870 kg/m^3^. The acoustic waves are detected by a passive ultrasonic array after propagating through a scattering layer. The scattering layer contains 40 scatterers with identical parameters compared to the ones in the ROI and the scatterers are randomly distributed. The scattering layer has a thickness of 20 mm and a concentration of 4 rods/cm^3^, corresponding to a frequency-averaged scattering mean free path of *l*_s_ = 20.9 ± 1.00 mm between 1.26 and 2.68 MHz^32^. The parameters for the surrounding medium are *c*_e_ = 1500 m/s and *ρ*_e_ = 1000 kg/m^3^, which approximate water or soft tissues. Therefore, the surrounding medium has seriously impedance mismatch with the scatterers, which leads to strong acoustic scattering. The passive ultrasonic transducer array has *N* = 101 elements with a pitch size of *w* = 0.5 mm. The distance between the array and the scattering layer is set as *a* = 40 mm and the signals are recorded with a sampling frequency of 20 MHz. We will concentrate on recovering the image of the ROI as this region is the furthest away from the array and the corresponding signals are worst because of the pollution by random acoustic scattering. The length of the time window ∆*t* is set to 5 μs, which promises that the direct waves within ROI are at the same time window^32^.

### Acquisition and processing of the signals

The acoustic waves are received by the array consisting of *N* ultrasonic transducers for imaging. The *N*-channel signals are recorded in a vector ${\bf{H}}(t)=[{H}_{1}(t),\mathrm{...},{H}_{n}(t),\mathrm{...},{H}_{N}(t)]$ and the vector can be converted to its frequency domain form as ${\bf{P}}(T,f)=[{P}_{1}(T,f),\mathrm{...},{P}_{n}(T,f),\mathrm{...},{P}_{N}(T,f)]$ with ${P}_{n}(T,f)={\int }_{T-{\rm{\Delta }}t/2}^{T+{\rm{\Delta }}t/{\rm{2}}}[{H}_{n}(\tau ){W}^{\ast }(\tau -T)]{e}^{-j2\pi f\tau }d\tau$, where *W*(*t*) is a window function. In the scattering medium, **P**(*T*, *f*) usually consists of two components, the direct wave **P**^D^(*T*, *f*  ) and the scattering wave **P**^S^(*T*, *f*  ). Based on the paraxial approximation, the element of the direct wave **P**^D^(*T*, *f*  ) that is from the source at (*X*, *Z*) can be written as

1 $${P}_{n}^{{\rm{D}}}(T,f)={A}_{0}\exp (\,jkZ)\exp \,[jk\frac{{({x}_{n}-X)}^{2}}{2Z}],$$

where *A*_0_ = *Z*^1/2^, *k* = 2π*f*/*c* is the wave number, *c* is the sound speed, *x*_*n*_ is the *x*-coordinate of the *n*-th transducer. The element of scattering wave **P**^S^(*T*, *f* ) is given by

2 $${P}_{n}^{{\rm{S}}}(T,f)\approx \sum _{l=1}^{L}[{A}_{l}\,\exp (jk{s}_{l})],$$

where *A*_*l*_ and *s*_*l*_ are the amplitude and phase corresponding to the *l*-th propagating path. Both *A*_*l*_ and *s*_*l*_ are random owing to the randomly distribution of the scatterers and the uncertainty of the scattering paths. Waves propagating through *L* scattering paths in the scattering medium constitute the scattering wave term. Then a response matrix **K**(*T*, *f* ) can be written as^31^

3 $${\bf{K}}={\bf{P}}{{\bf{P}}}^{{\rm{T}}}=\mathop{\underbrace{{{\bf{P}}}^{{\rm{D}}}{({{\bf{P}}}^{{\rm{D}}})}^{{\rm{T}}}}}\limits_{{\rm{Coherence}},{{\bf{K}}}^{{\rm{C}}}}+\mathop{\underbrace{[{{\bf{P}}}^{{\rm{D}}}{({{\bf{P}}}^{{\rm{S}}})}^{{\rm{T}}}+{{\bf{P}}}^{{\rm{S}}}{({{\bf{P}}}^{{\rm{D}}})}^{{\rm{T}}}+{{\bf{P}}}^{{\rm{S}}}{({{\bf{P}}}^{{\rm{S}}})}^{{\rm{T}}}]}}\limits_{{\rm{Random}},{{\bf{K}}}^{{\rm{R}}}}.$$

It is worth noticing that all terms in Eq. (3) containing **P**^S^ are random due to the random nature of multiple scatterings. We recorded them as **K**^R^. The remaining term recorded as **K**^C^ is independent of the scatterer distribution and the scattering paths. Its element can be given as

4 $${k}_{m,n}^{{\rm{C}}}={A}_{0}^{2}\exp (j2kZ)\exp [jk\frac{{({x}_{n}-X)}^{2}+{({x}_{m}-X)}^{2}}{2Z}].$$

A deterministic relation of the phase between the elements ${k}_{m,n}^{{\rm{C}}}$ exists along the antidiagonal,

5 $${\phi }_{q}={k}_{m-q,m+q}^{{\rm{C}}}/{k}_{m,m}^{{\rm{C}}}=\exp [jk{(qw)}^{2}\,/Z].$$

**Figure 2:**
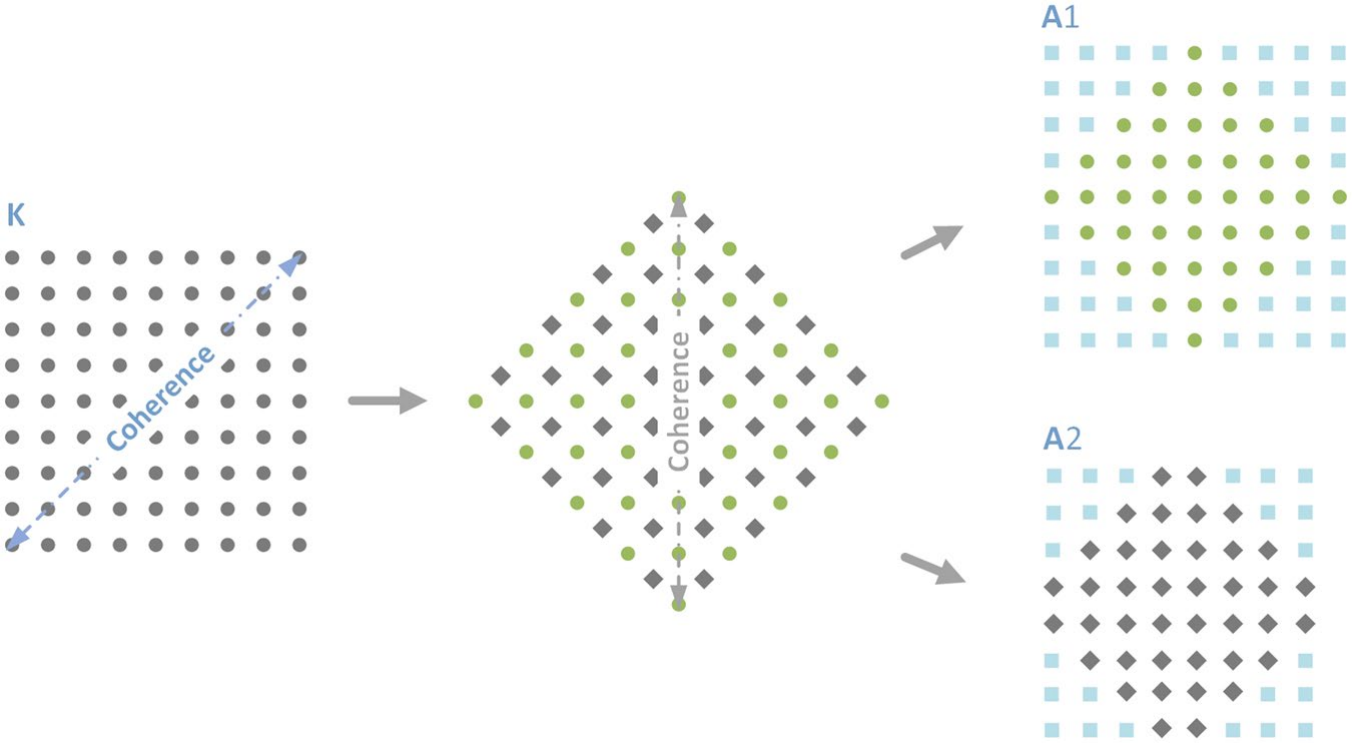
Principle of the matrix rotation by taking the example of a matrix K with a dimension *N* = 9. The gray circles donate the whole elements of **K**. By rotating the matrix 45 degrees, the coherence of the direct waves appears along the columns and all the elements are divided into the green circle part and gray diamond part according to the symmetry characteristics. Finally, these two parts are extended into two new matrices **A**1 and **A**2. The value of the blue square elements is 0.

That is, the phase difference between two matrix elements in the same antidiagonal only depends on the channel spacing *qw* and is irrelevant to the acoustic sources or scattering paths. Direct waves represent a particular coherence on the antidiagonals of the matrix. This coherent property along the antidiagonals in the matrix form allows us to extract direct waves from the scattering background by applying a correlation filter^31^. Aiming at breaking through the limitations of previous study^31^, we have proposed the following process of matrix filtering:

*Step* 1: Two new matrices **A**1 and **A**2 are built by rotating the matrix **K**(*T*, *f* ) by 45 degrees. As shown in Fig. 2, the original matrix **K** is rotated by 45° counterclockwise and the coherence of direct waves occurs on the columns as a consequence. Then we can divide the element into two parts based on their symmetry characteristic. For the green circle part, the center element of each line falls on the axis of symmetry. When it comes to the gray diamond part, the axis of symmetry is in the middle of two elements. Finally, we separate the two parts and expand them into two new matrices **A**1 and **A**2, respectively. The value of the blue square elements in Fig. 2 is 0. The element *a*1_*u*,*v*_ and *a*2_*u*,*v*_ of matrices **A**1 and **A**2 can be related to the element *k*_*m*,*n*_ of matrix **K** by

6 $$\{\begin{array}{ccccccc}a{1}_{u,v} & = & {k}_{m,n}, & {\rm{as}} & u+v\in (\frac{N+1}{2},\frac{3N+3}{2}) & {\rm{and}} & v-u\in (-\frac{N+1}{2},\frac{N+1}{2})\\ \,a{1}_{u,v} & = & 0, & {\rm{as}} & u+v\notin (\frac{N+1}{2},\frac{3N+3}{2}) & {\rm{or}} & v-u\notin (-\frac{N+1}{2},\frac{N+1}{2})\end{array}$$

7 $$\{\begin{array}{ccccccc}a{2}_{u,v} & = & {k}_{m,n}, & {\rm{as}} & u+v\in (\frac{N-1}{2},\frac{3N+1}{2}) & {\rm{and}} & v-u\in (-\frac{N+1}{2},\frac{N+1}{2})\\ \,a{2}_{u,v} & = & 0, & {\rm{as}} & u+v\notin (\frac{N-1}{2},\frac{3N+1}{2}) & {\rm{or}} & v-u\notin (-\frac{N+1}{2},\frac{N+1}{2})\end{array}$$

where *m* = *u* + *v* − (*N* + 1)/2 and *n* = *v* − *u* + (*N* + 1)/2. The dimensions of matrices **A**1 and **A**2 are *N* and *N* − 1, respectively. In the following discussion, we will no longer make the difference between matrices **A**1 and **A**2 because they are filtered in the same way. They will be called indifferently **A**. The dimension of the matrix **A** is marked as *N*_A_. The coherence of direct waves is transferred to the columns of **A** after the rotation.

*Step* 2: A filtering matrix **F** = **CC**^†^ is constructed to extract coherence part from the matrix **A**, where $${\bf{C}}=[\exp (jk{y}_{1}^{2}/2Z),\,\mathrm{...},\exp (jk{y}_{u}^{2}/2Z),\mathrm{...},\exp (jk{y}_{{N}_{A}}^{2}/2Z)]$$ with *u* = 1, 2, …, *N*_*A*_ and $${y}_{u}=[({x}_{m}+{x}_{n})+$$$$({N}_{A}-1)w]/\sqrt{2}$$ is the characteristic space of direct waves. We can obtain the filtered matrix **A**^F^ according to the formula

8 $${{\bf{A}}}^{{\rm{F}}}={\bf{F}}{\bf{A}}={\bf{F}}({{\bf{A}}}^{{\rm{C}}}+{{\bf{A}}}^{{\rm{R}}})={\bf{F}}{{\bf{A}}}^{{\rm{C}}}+{\bf{F}}{{\bf{A}}}^{{\rm{R}}}$$

The first term in the right of the formula **FA**^C^ = **A**^C^ indicates that the coherence part remains itself after the filtering process. In contrast, the components that are orthogonal to the characteristic space of the direct waves will be filtered out. For **A**1, the filtering process will decrease the scattering contribution by a factor $\sqrt{(N+1)/2}$ since each column of **A**1 contains (*N* + 1)/2 independent coefficients considering its symmetry^25^.

*Step* 3: A filtered matrix **K**^F^ with a change of coordinates back to the original system can be obtained by applying the coordinate inversion to **A**^F^. The matrix element is given by

9 $$\begin{array}{llll}{k}_{m,n}^{{\rm{F}}} & = & a{1}_{(m-n)/2+(N+3)/4,(m+n)/2}^{{\rm{F}}}, & {\rm{if}}\,(m-n)\,{\rm{is}}\,{\rm{even}}{\rm{.}}\\ {k}_{m,n}^{{\rm{F}}} & = & a{2}_{(m-n-1)/2+(N+3)/4,(m+n-1)/2}^{{\rm{F}}}, & {\rm{if}}\,(m-n)\,{\rm{is}}\,{\rm{odd}}.\end{array}$$

The filtered matrix **K**^F^ has the same dimension *N* as the original detected matrix **K**. Before and after filtering, the dimensions of the matrix remain unchanged. No elements are excluded by the filter.

*Step* 4: A time reversal operator can be applied to recover the image of acoustic sources from the filtered matrix **K**^F^. Each acoustic source is associated with one non-zero singular of matrix **K**^F^. The backpropagated image of the *i*-th singular value ${{\bf{I}}}^{i}(Z=cT,f)=\{{I}_{1}^{i},\,{I}_{2}^{i},\,\mathrm{...}\,,\,{I}_{N}^{i}\}$ is ${{\bf{I}}}^{i}(Z,f)={\lambda }_{i}^{{\rm{F}}}|{\bf{G}}\ast {{\bf{V}}}_{i}^{{\rm{F}}}|$, where **G** is the propagating operator in a homogeneous media, ${\lambda }_{i}^{{\rm{F}}}$ is the *i*-th singular value of **K**^F^, and ${{\bf{V}}}_{i}^{{\rm{F}}}$ is the normalized singular vector corresponding to ${\lambda }_{i}^{{\rm{F}}}$. The component of **G** is expressed as ${g}_{m,n}=\exp (jk{r}_{m,n})/\sqrt{{r}_{m,n}}$ with *r*_*m*,*n*_ the distance between the *n*-th transducer and the *m*-th point in the focal plane. ${\lambda }_{i}^{{\rm{F}}}$ and ${{\bf{V}}}_{i}^{{\rm{F}}}$ can be obtained by applying the singular value decomposition to the matrix **K**^F^(*T*, *f* ) = **U**^F^**Λ**^F^**V**^F†^. The image in the focal plane Z = *cT* can be achieved by ${{\bf{I}}}^{{\rm{F}}}=\sum _{i=1}^{M}{{\bf{I}}}^{i}$, where *M* is the total number of the non-zero singular values used for imaging. ${{\bf{I}}}^{{\rm{F}}}=\{{I}_{1}^{{\rm{F}}},\,{I}_{2}^{{\rm{F}}},\,\mathrm{...}\,,\,{I}_{N}^{{\rm{F}}}\}$ represents the pixel values of the points in the focal plane *Z* = c*T*.

*Step* 5: The image distortion and false contrast due to the limited-view detection are compensated by

10 $${I}_{m}^{{\rm{U}}}=\frac{{I}_{m}^{{\rm{F}}}}{{I}_{m}^{{\rm{R}}}},$$

where ${I}_{m}^{{\rm{U}}}$ is the ultimate pixel value of the *m*-th point in the focal plane *Z = *c*T* after compensating and ${I}_{m}^{{\rm{R}}}$ is the corresponding location factor calculated for compensating. Since the array does not surround the imaging region completely, the limited-view detection will cause the false contrast and distortion. Considering a single point source located at the *m*-th point in the focal plane *Z* = c*T* without the scattering layer, the detected signal should be ${\bf{P}}=({g}_{m,1},{g}_{m,2},\,\mathrm{...}\,,{g}_{m,N})$. The response matrix **K** = **PP**^T^ is equal to the filtered matrix **K**^F^, i.e., **K**^F^ = **K** if there is no scattering layer. The singular vector of **K**^F^ is ${\bf{V}}\propto ({g}_{m,1},{g}_{m,2},\,\mathrm{...}\,,{g}_{m,N})$ and the imaging intensity of the point source is

11 $${I}_{m}^{{\rm{R}}}=\Vert {g}_{m,1}^{\ast }{g}_{m,1}+{g}_{m,2}^{\ast }{g}_{m,2}+\cdots +{g}_{m,N}^{\ast }{g}_{m,N}\Vert =\sum _{n=1}^{N}\frac{1}{{r}_{m,n}}.$$

Equation (11) indicates that the imaging intensity is also related to the position of the acoustic source besides of its intensity, which is said that the false contrast is induced by the limited view detection. Therefore, the image can be corrected by compensating the location factor ${I}_{m}^{{\rm{R}}}$ as Eqs (10) and (11).

### Imaging results and data analysis

**Figure 3:**
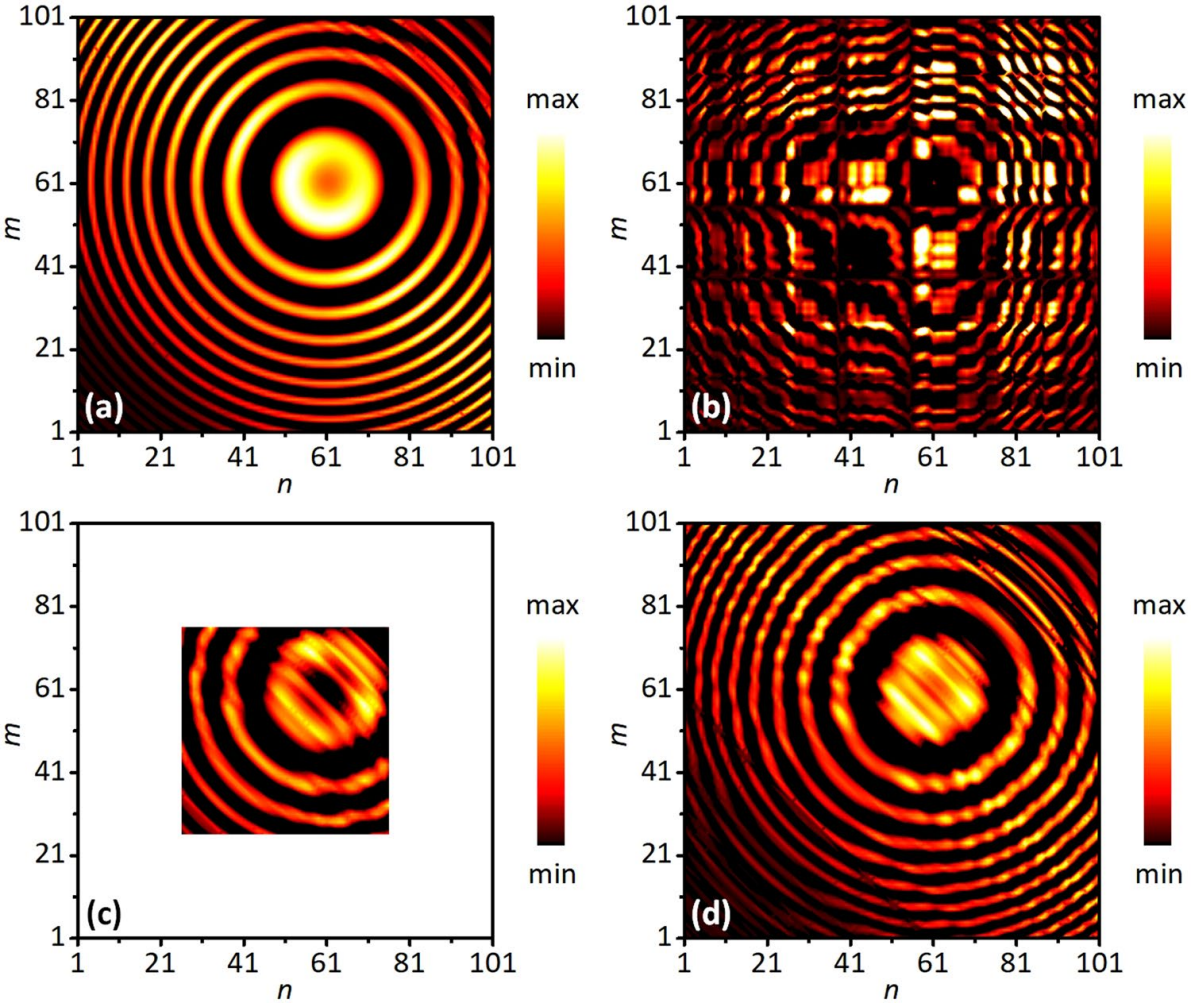
Results in a matrix formalism of the signals at time *T* = 58 μs and frequency *f* = 2.0 MHz. (**a**) Real part of **K** without the scattering layer. (**b**) Real part of **K** with the scattering layer. (**c**) Real part of **K**^F^ given by the filtered time reversal (FTR) method. (**d**) Real part of **K**^F^ given by the correlation full-matrix filter (CFMF) method.

Figure 3 illustrates the action of the matrix filtering on the recorded signals at frequency *f* = 2.0 MHz and time *T* = 58 μs, corresponding to the source 2 in the time window in Fig. 1. For reference, Fig. 3(a) shows the matrix **K** when the scattering layer is absent and only one acoustic source exists. The regular pattern implies the deterministic coherence of the matrix **K**. When the scattering layer is placed between the ROI and the array, the scattering waves break the deterministic coherence of the matrix **K**, as shown in Fig. 3(b). Figure 3(c,d) present the filtered matrix **K**^F^ obtained by the filtered time reversal (FTR) method^31^ and the proposed correlation full-matrix filter (CFMF) method, respectively. As shown, both methods can recover the coherent characteristics and reduce scattering components. However, the dimension of **K**^F^ obtained by the FTR method (Fig. 3(c)) is only half of the reference matrix **K** in Fig. 3(a). It means that part of the detected information is lost. On contrast, **K**^F^ obtained by the CFMF method (Fig. 3(d)) is highly consistent with the reference matrix **K** (Fig. 3(a)). The filtered matrix not only reproduces the coherent characteristics but also maintains the matrix dimension. It preserves all useful information of the direct wave but cleans up disturbance from the scattering waves.

**Figure 4:**
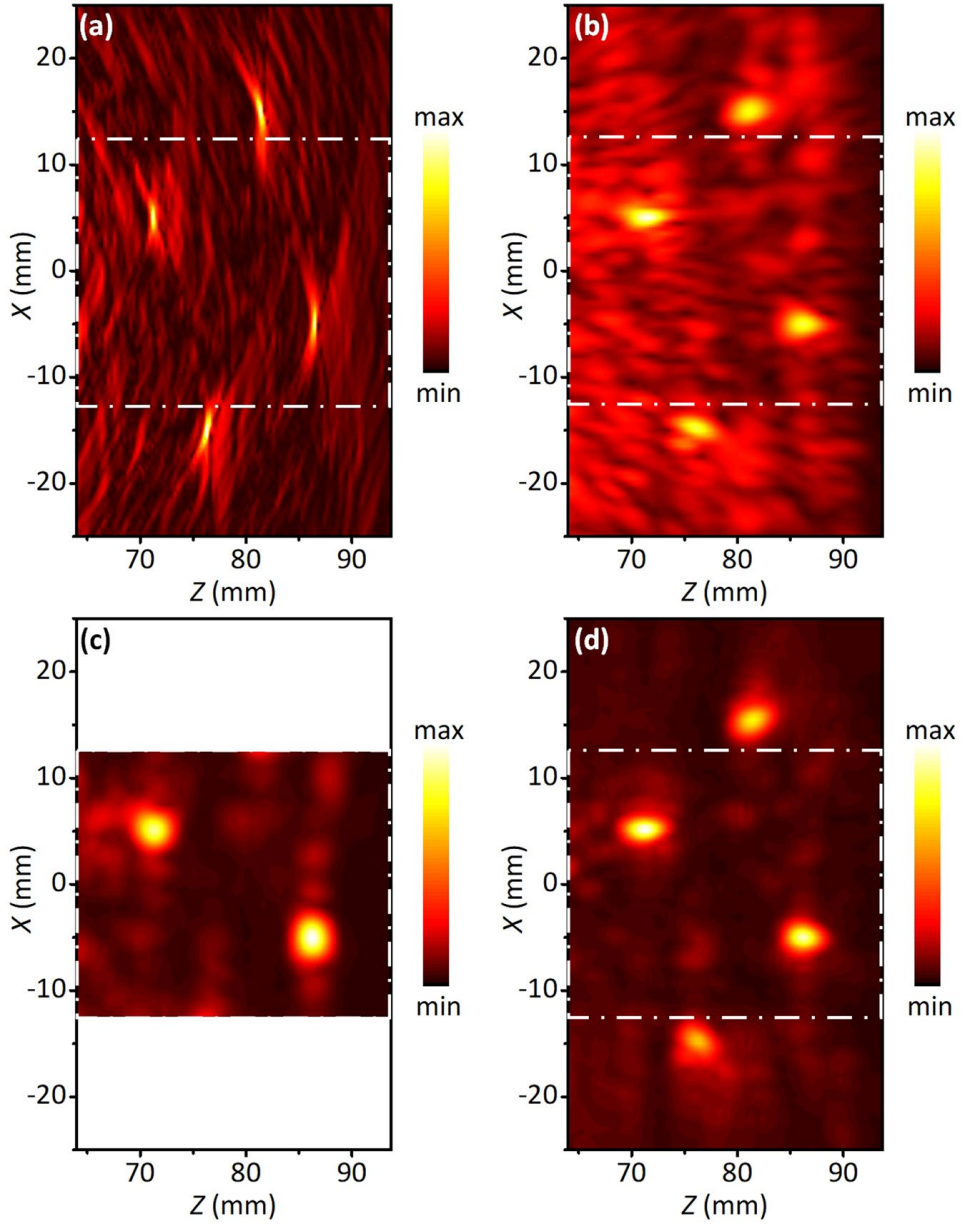
Reconstructed images of the ROI behind an acoustically scattering layer. (**a**) Image reconstructed by the delay-and-sum (DAS) beamforming method. (**b**) Image reconstructed by the time reversal operator without filtering. (**c**) Image reconstructed by the FTR method. (**d**) The final image after applying the CFMF method. The CFMF method significantly reduce the background noise (**d**), in comparison with the other methods (**a**–**c**).

Figure 4 illustrates the images behind the scattering layer recovered by the conventional delay-and-sum (DAS) method, the time reversal method, the FTR method, and the current method, respectively. Both the DAS method (Fig. 4(a)) and the time reversal method (Fig. 4(b)) can recover the images of the ROI. However, low contrast and strong speckles causing by the strong scattering appear in the background. The FTR method (Fig. 4(c)) cleans the speckles in background very well since the scattering waves are reduced. However, it only obtains the partial image of the ROI, because the filtering process reduces the matrix dimension and cause the absence of the detected information. Figure 4(d) shows the imaging result obtained by the CFMF method. The image has a good contrast. The background noise has been significantly reduced after filtering. Additionally, the full image of the ROI is obtained since the filtered matrix has the same dimension as the original matrix and the direct wave is fully utilized. Visually, the CFMF method provides much better images behind a strong scattering layer, in comparison with the traditional methods.

**Figure 5:**
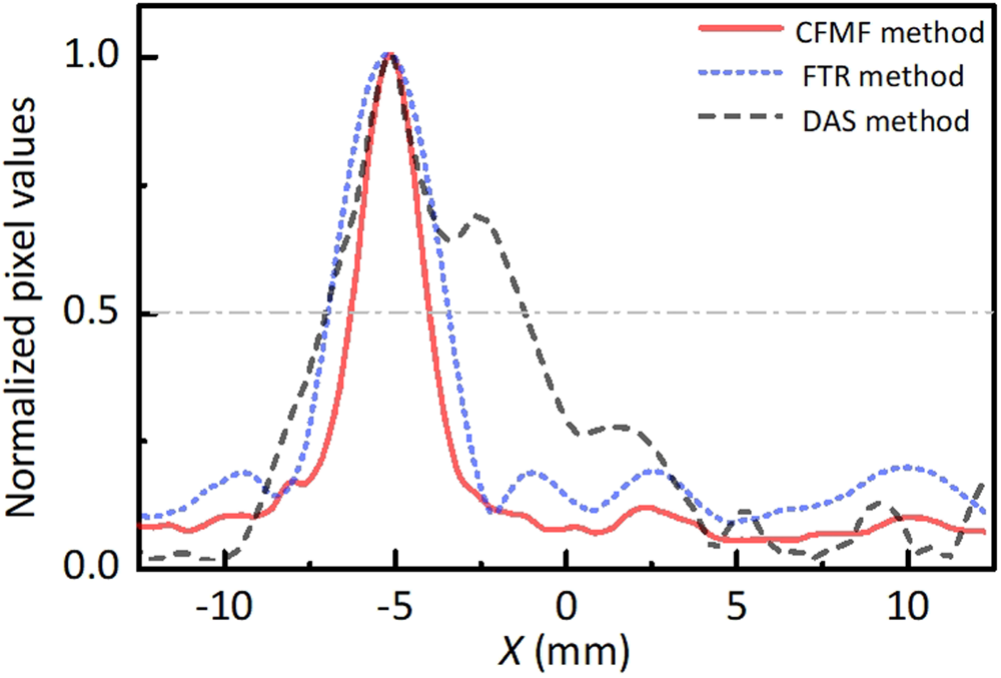
Image profile of the target obtained by three different imaging methods. Normalized pixel values in the focal plane at a depth *Z* = 87 mm (corresponding to source 2) of the CFMF method (red solid line), the FTR method (blue dotted line) and the traditional DAS method (black dashed line). The full width at half maximum (FWHM) of each method is measured by the grey dotted line. The CFMF method provides the smallest FWHM, which implies the best resolution.

Figure 5 shows the profile of images along the focal plane at the depth *Z* = 87 mm, as shown by the time window in Fig. 1. These curves correspond to the source 2 and are normalized to their maximum. The image recovered by the DAS method (black dashed line) has the largest full-width at half-maximum (FWHM) of 5.57 mm because of the serious imaging distortion and the strong background noise. It implies a poor resolution and contrast. The FTR method (blue dotted line) has improved the FWHM to 3.54 mm. The CFMF method (red solid line) has the narrowest half-maximum width of 2.28 mm and the lowest background noise. It is said that the CFMF method not only enlarges the image region, but also further reduces the speckles and improves the imaging resolution in comparison with the FTR method.

**Figure 6:**
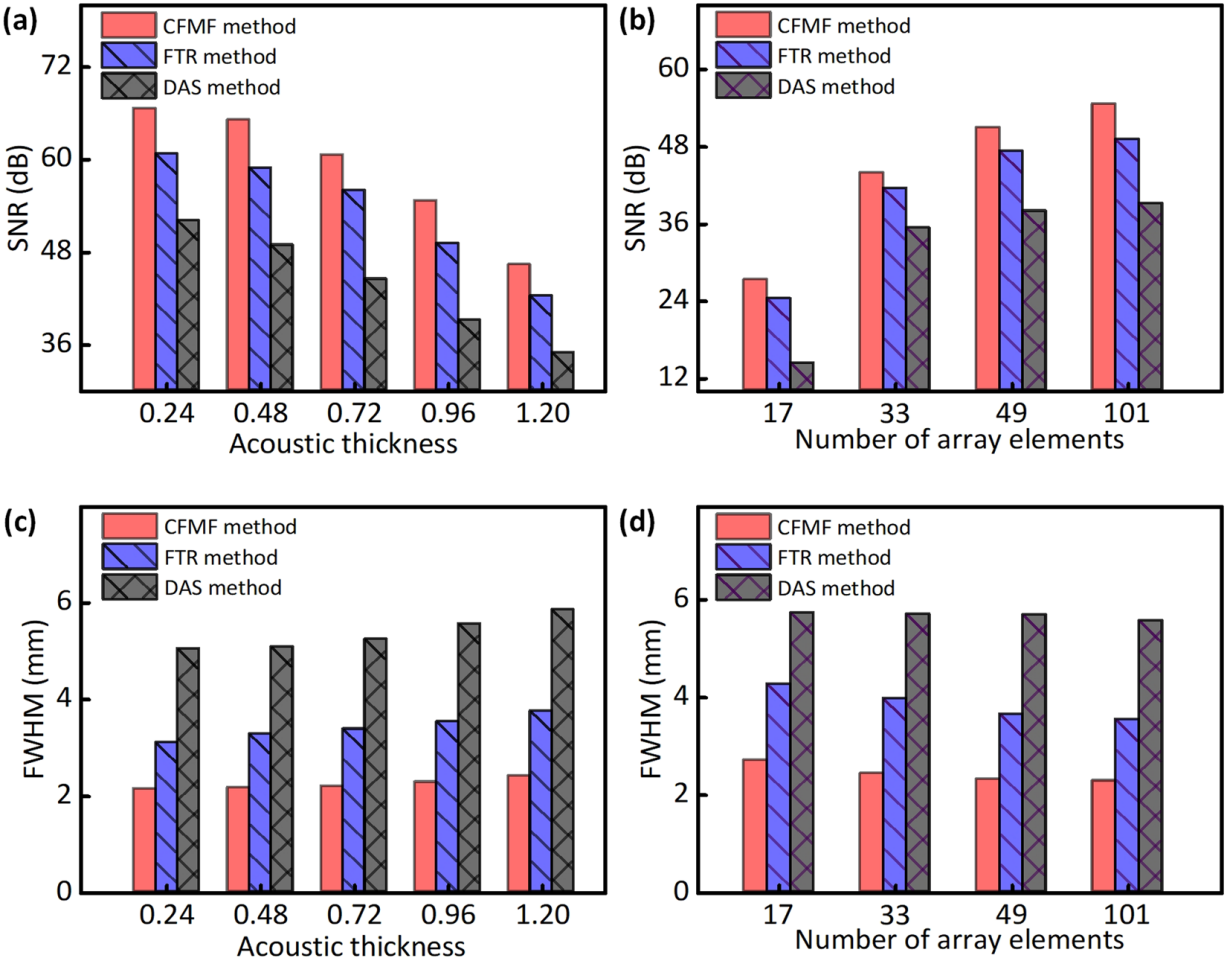
The comparison of the signal-to-noise ratio (SNR) and the FWHM between three different methods. (**a**) The SNR of three methods in the case of different scattering layer. (**b**) The SNR of three methods in the case of different array element number. (**c**) The average FWHM of three methods in the case of different scattering layer. (**d**) The average FWHM of three methods in the case of different array element number.

Figure 6 compares the performances of the three methods under different scattering conditions. The signal-to-noise ratio (SNR) and the average FWHM are utilized to quantify the imaging quality. For the sake of comparison, the SNR of the same region within – 12.5 mm ≤ X ≤ 12.5 mm and 63 mm ≤ Z ≤ 92.7 mm is calculated. Considering the actual size and position of the acoustic sources, we defined the area inside the two circles with a diameter of 0.8 mm at the locations of the sources 2 and 4 as the signal area. For comparison, we defined the area outside the two circles and inside the rectangle labelled by the white dotted lines in Fig. 4 as the background area. The ratio of the mean signal intensity in the signal area and the mean noise intensity in the background area is defined as SNR. The FWHM for each acoustic source of the images is estimated by using the same way discussed in Fig. 5. A smaller FWHM and lower SNR imply the better quality of the image.

Figure 6(a,c) compare the imaging qualities in the case of different scattering layers, where the number of the scatterers in the scattering layer is increased from 10 to 50. As the scatterer number increases, the frequency-averaged scattering mean free paths are reduced from 83.75 ± 4.00 mm to 16.75 ± 0.80 mm with a corresponding range of frequency from 1.26 MHz to 2.68 MHz. Acoustic thickness is the ratio of the thickness of the scattering layer and the frequency-averaged scattering mean free path. As shown in Fig. 6(a), all the three methods can provide high SNR when the scattering is weak. When the acoustic scattering is stronger, the image obtained by the CFMF method still has good SNR and always remains the highest SNR among the three methods. For the DAS method, the FWHM becomes larger as the acoustic thickness of the scattering layer increases (Fig. 6(c)). Results of the FTR method show the same trend but the changes are smaller. When it comes to the CFMF method, the acoustic thickness has little impact on the FWHM and the imaging performance is more stable. Furthermore, the targets in the images obtained by the CFMF method have the smallest FWHM, in comparison with the FTR method and the DAS method. Figure 6(b,d) examine the imaging qualities when the number of the array elements is reduced from 101 to 17, where the number of scatterers is 40. Here, the length of the array remains the same. Fewer array elements means a larger array pitch. The image qualities are improved as the transducer number increases. The CFMF method always has the best SNR and FWHM in comparison with the other methods. Moreover, the CFMF method can still have good performances even when the number of transducers is reduced to 17. These results suggest the proposed method can always provide the best images with the lowest speckles and the best resolution under different scattering situations or detection situations, comparing to the other methods.

### Experimental set-up and imaging results

**Figure 7:**
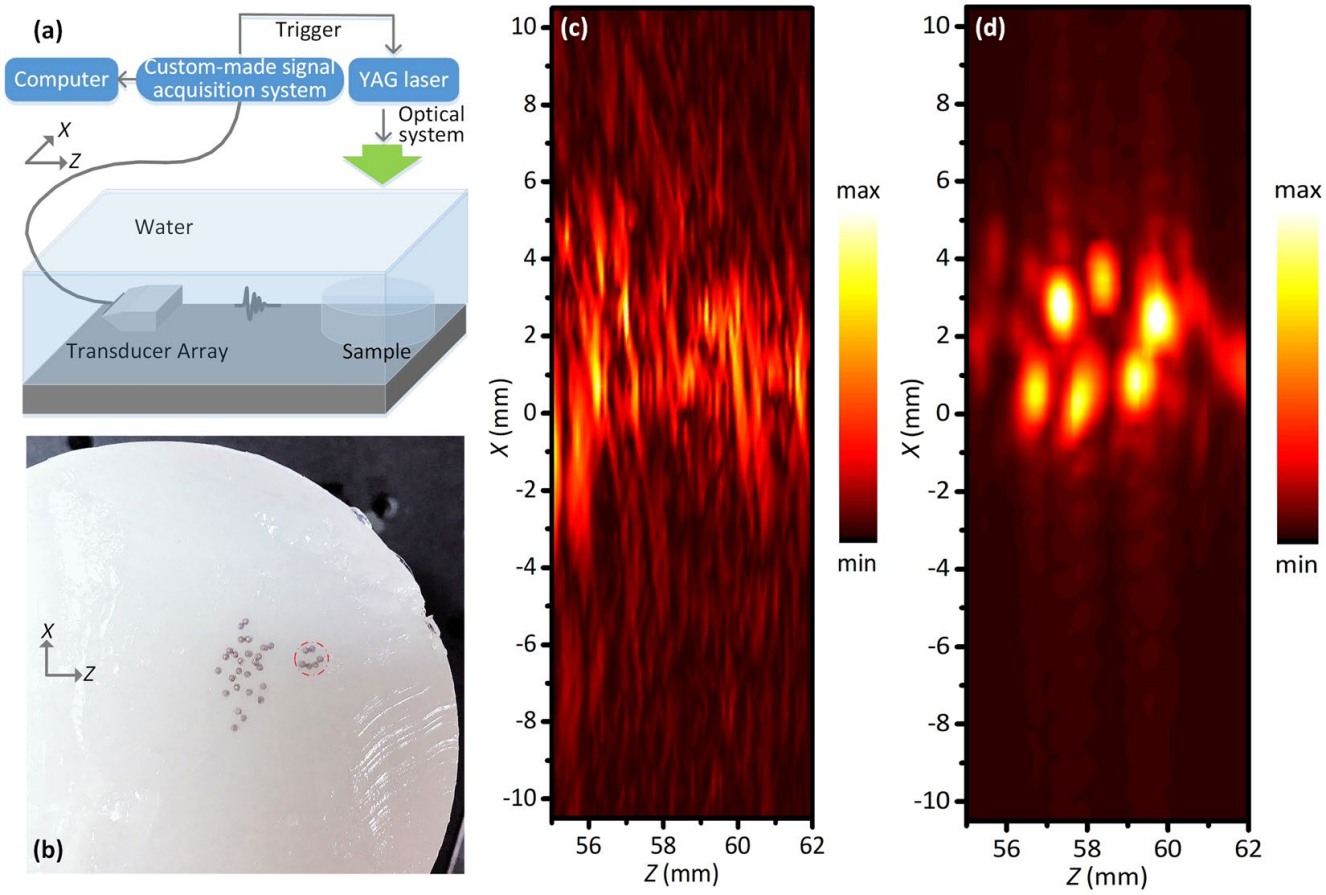
Comparison of experimental results. (**a**) Schematic of experimental set-up. (**b**) Photo of the sample used in the experiment. (**c**) Image reconstructed by the DAS method. (**d**) Image reconstructed by the CFMF method.

Figure 7(a) illustrates the schematic diagram of the experimental system. Irradiated by the pulse laser with a wavelength of 532 nm and a pulse width of 8 ns, the targets in the sample emit ultrasonic waves owing to photoacoustic effect. The waves will propagate through the scattering layer and then be picked up by the 64-channel transducer array (7.5MhzL46, Haiying Inc.) with a central frequency of 7.5 MHz. The signals are sampled and recorded by the 64-channel custom-made signal acquisition system and are finally stored by the PC system. The sampling frequency is 32 MHz. As shown in Fig. 7(b), six steel balls are arranged in a smiley face pattern. A scattering layer with a thickness of 10 mm is placed between the imaging region and the transducer array. 26 steel balls are distributed randomly within the scattering layer. The diameter of all these steel balls is 0.8 mm and all the balls are embedded in the agar for fixing, and the speed of sound of the agar is measured as 1460 m/s at 25 °C.

For comparison, the images recovered by DAS method and CFMF method are shown in Fig. 7(c,d). The pattern recovered by the DAS method cannot be clearly distinguished. Strong background noise and graphic distortion lead to the low contrast of the image. When it comes to the imaging results of CFMF method, we can get the clearly smiley face pattern as well as some important details, including the total number and the distribution of steel balls. It can be concluded that the improvement of imaging quality by the CFMF method is also significant in the real photoacoustic application, which is consistent with previous theoretical predictions. For the sake of quantitatively analyzing the image quality, including the SNR and the FWHM, we have done the simulations and experiments in the case of point sources. To further confirm the application of the proposed method, we have added the simulation results of the vessel shape sources to the supplemental material. The CFMF method is also applicable and have better performance in the case of vessel shape sources (see Supplementary Note. 1).

## Discussion

We present a full-matrix filter based on the correlation of direct waves to overcome the obstacles of photoacoustic imaging in the inhomogeneous media. In comparison to our previous studies, two major improvements have been achieved. Firstly, we adopt a full-matrix filtering process, which can effectively extract the coherence components in the detected signal and preserve all the useful information. Secondly, a location factor ${I}_{m}^{{\rm{R}}}$ is considered in the time reversal operator to compensate for the image distortion and false contrast. As a result, the proposed approach can perform high quality imaging with higher image SNR, better resolution, and wider imaging area, in comparison with the DAS method and the FTR method. This work might be valuable in studying the physics of the interaction of sound in the complex media. Moreover, the proposed approach can be applied to improve the quality of photoacoustic imaging for inhomogeneous biological tissues which could be seen as linear combinations of point sources that are not independent.

## Methods

### Numerical simulations

Throughout the paper, Finite Element Method based on commercial software COMSOL Multiphysics^TM^ 5.3a is employed for the simulations. Impedance boundary condition is imposed on the outer boundaries of simulated domain to eliminate the interference from reflected waves.

## Electronic supplementary material

Supplementary Material

## Data Availability

The datasets generated during and/or analyzed during the current study are available from the corresponding author on reasonable request.
