# Supplementary material for: Imaging acoustic sources through scattering media by using a correlation full-matrix filter
Source: Sci Rep. 2018 Oct 23;8:15611. doi: 10.1038/s41598-018-34039-w (PMC6199323; doi:10.1038/s41598-018-34039-w)
Supplement: Supplementary file 1 — Supplementary Material [file 41598_2018_34039_MOESM1_ESM.pdf]

Supplementary Material for the paper

"Imaging acoustic sources through scattering media by using a correlation full-matrix filter"

Wei Rui[1], Chao Tao[1,2], Xiaojun Liu[1,3]

[1] Laboratory of Modern Acoustics, Department of Physics, Collaborative Innovation Center of Advanced Microstructures, Nanjing University, Nanjing, 210093, China

[2] Shenzhen Research Institute of Nanjing University, Shenzhen, 51800, China

[3] Email: liuxiaojun@nju.edu.cn

Correspondence and requests for materials should be addressed to C.T. (email: taochao@nju.edu.cn)

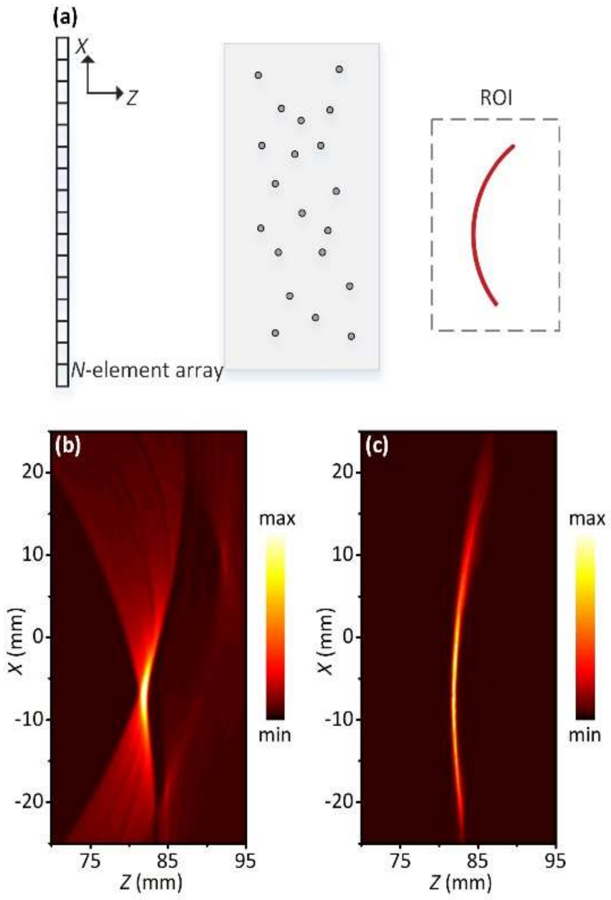

Figure S1. Simulation results in the case of vessel shape sources (**a**) Schematic of the simulation model. (**b**) Image reconstructed by DAS method. (**c**) Image reconstructed by CFMF method.

Note 1. Simulation results in the case of vessel shape sources

As shown in Fig. S1(a), a vessel shape source with a width of 0.5 mm is located in the ROI behind the scattering layer. The number of the scatterers in the scattering layer is 20 and the frequency-averaged scattering mean free path is 41.88 ± 2.00 mm. The medium in the vessel shape source is set as water, the same as the environmental medium. Figure S1(b) gives the image recovered by the DAS method, in which the shape of the source is severely distorted as a result of the limited-view scanning and the scattering waves. On contrary, the image given by the CFMF method [Fig. S1(c)] restores the general shape of the vessel with a high contrast. The simulation results show that the CFMF method is also applicable in the case of source which could be seen as linear combinations of point sources that are not independent.
